# Taxonomic resolution of the ribosomal RNA operon in bacteria: implications for its use with long-read sequencing

**DOI:** 10.1093/nargab/lqz016

**Published:** 2019-11-14

**Authors:** Leonardo de Oliveira Martins, Andrew J Page, Alison E Mather, Ian G Charles

**Affiliations:** 1 Quadram Institute Bioscience, Norwich Research Park, Norwich, NR4 7UQ, UK; 2 Faculty of Medicine and Health Sciences, University of East Anglia, Norwich, NR4 7TJ, UK

## Abstract

DNA barcoding through the use of amplified regions of the ribosomal operon, such as the 16S gene, is a routine method to gain an overview of the microbial taxonomic diversity within a sample without the need to isolate and culture the microbes present. However, bacterial cells usually have multiple copies of this ribosomal operon, and choosing the ‘wrong’ copy could provide a misleading species classification. While this presents less of a problem for well-characterized organisms with large sequence databases to interrogate, it is a significant challenge for lesser known organisms with unknown copy number and diversity. Using the entire length of the ribosomal operon, which encompasses the 16S, 23S, 5S and internal transcribed spacer regions, should provide greater taxonomic resolution but has not been well explored. Here, we use publicly available reference genomes and explore the theoretical boundaries when using concatenated genes and the full-length ribosomal operons, which has been made possible by the development and uptake of long-read sequencing technologies. We quantify the issues of both copy choice and operon length in a phylogenetic context to demonstrate that longer regions improve the phylogenetic signal while maintaining taxonomic accuracy.

## INTRODUCTION

Microbes are the most numerous organisms on the planet, and although some are of great importance to our health and well-being, we do not understand the full diversity of microbes present (1). This is compounded by the fact that only a small number of bacterial species can currently be cultured. Knowing the diversity of the microbial population of an environment, the microbiota, can allow us to begin to understand their relevance, such as measuring the impact of changes in diet or drugs on the gut microbiota (2,3). Short-read shotgun sequencing of amplified regions of 16S, 23S ribosomal RNA genes and the internal transcribed spacer (ITS) region have become a cheap, routine and direct way to gain a high-level understanding of microbial taxonomic diversity within complex samples, such as feces or soil, without requiring culturing of microbes (4,5). For example, evaluating the short hypervariable regions of the 16S gene gives family-level taxonomic resolution, distinguishing between the common *Staphylococcal* and *Streptococcal* pathogens (6).

This approach has limitations as it is expected to detect not only just previously characterized microbes but also those for which we have very little knowledge, using genome regions that are theorized to exist in all microbes. There can be multiple copies of the ribosomal operon within a bacterium, and the variation between operons within a single bacterium can exceed the variation between different species (7). For example, *Salmonella enterica* has 7 ribosomal operons while *Bacillus thuringiensis* has 17 rRNA operons. Although there are techniques capable of discriminating between paralog copies, reducing chimerism and primer bias (8), sequencing of different operons even within the same species can therefore give conflicting taxonomic identification. Linking the phylogenetic signal to these short amplified markers can be challenging, so it is important to understand the limitations and potential of current technologies.

Full-length 16S RNA can be recovered using short-read platforms (9), but the 16S-ITS-23S region of the rRNA operon has four times the variability of the 16S region alone, and can be used to classify sequences taxonomically, even at the strain level in certain cases (10). This is more accurate for well-characterized species where large numbers of genome sequences, the majority of which are short-read sequences, allow a greater understanding of ribosomal operon diversity. However, the best resolution of this variable operon is obtained through complete, contiguated genome sequences; long-read sequencing provides reads that can span the entire operon. As the cost of long-read sequencing continues to fall, it is set to replace short-read sequencing. Despite this, the taxonomic resolution achievable using long-read sequencing of the entire length of the ribosomal operon is not well explored. Here, we use a set of 691 publicly available complete reference genomes as our data set, explore different combinations and lengths of a representative ribosomal operon and evaluate their utility in providing taxonomic resolution. We also studied qualitatively all copies of the full-length ribosomal operon from a set of 194 *Staphylococcus* and *Pseudomonas* genomes. These experiments allow us to define the theoretical boundaries when using the full-length ribosomal operon for population diversity experiments. We use a phylogenetic rationale to infer the effect of rRNA gene choice in taxonomic classification, and we demonstrate that using a longer operon allows for an increase in both phylogenetic signal and classification accuracy.

## MATERIALS AND METHODS

### Single copy data sets

For analysis of single-copy data sets, we downloaded all complete bacterial assemblies from RefSeq (accessed on 15 February 2019) of selected Gram-positive and Gram-negative bacteria genera. We used the Genome Taxonomy Database (GTDB) both to provide the binomial nomenclature and to validate the sequences, since only curated genomes are included in GTDB (11). We analyzed all those with more than four strains from the same species, while also down-sampling over-represented ones. The genera were arbitrarily selected, based on pathogenic importance and availability, and comprised *Klebsiella*, *Enterococcus*, *Escherichia*, *Pseudomonas*, *Staphylococcus* and *Streptococcus*. For species with >32 strains, we assigned a probability of being chosen inversely proportional to their representativity in the database, such that we had, on average, 32 strains in the final data set. The final data set comprised 691 genomes. When a strain contained several copies of the same ribosomal RNA gene, we chose the longest gene to provide a single sequence for phylogenetic analysis. The sequence lengths, in base pairs (bp), were 1550 ± 32 for the 16S gene, 2908 ± 89 for the 23S gene and 116 ± 1 for the 5S gene. The related statistics for all genomes, including the number of copies per genome, can be found in the Supplementary Tables.

The hypervariable regions chosen where the v1+v2 and the v3+v4 segments of the 16S gene. They were found by similarity search using the sequence pair *AGAGTTTGATCCTGGCTCAG* and *ACTCCTACGGGAGGCAGCA* as flanking regions for 16Sv1v2, and sequences *CCTACGGGAGGCAGCAG* and *ATTAGAWACCCBDGTAGTCC* as flanking the segment 16Sv3v4. These sequences represent standard primers for these hypervariable regions (12), and the 16S regions mapped between them were included. If a particular primer is missing, its location was predicted using the other primers, or in the worst case the region was conservatively predicted using its relative location (6): for a 16S gene of any size the first 24% of the sites were chosen as segment 16Sv1v2 and the sites comprising the interval between 24% and 55% were assigned to segment 16Sv3v4. These, as well as the single genes 16S, 23S and 5S, were aligned independently, encompassing multiple common genomic regions used for taxonomic classification. Furthermore, the multi-locus data sets like 16S+23S and others were created by concatenating the previously aligned single-locus genes.

In real-world metagenomic data sets, it is not always possible to unambiguously distinguish between copies of the operon within a single strain because algorithms essentially create a chimeric or consensus sequence between all the copies. We simulated this scenario by calculating the consensus sequence between all copies, but then used the IUPAC ambiguity code rather than calling the ambiguity code N when the most frequent base cannot be accessed. This allowed us to preserve more information than would have been possible using typical methods, which mistake duplicates along the genome with polymorphism within a single copy. In terms of the tree silhouette score, this creation of consensus sequences did not affect the results when compared to the longest sequence, and are therefore not shown.

### Evaluation—tree silhouette score and monophyly score

We implemented both tree silhouette scores and monophyly scores for this study. The silhouette score is a measure of how close a sample is to others from the same cluster (species or genera, in our case), while at the same time how far away it is from samples from other clusters. It is commonly used in cluster analysis to define the optimal number of clusters. The silhouette score of strain *i* from cluster (species) *K* is defined as:

$$\begin{equation*}{s_i} = \frac{{d({i,J}) - d({i,K})}}{{MAX[ {d({i,J}),\,d({i,K})}]}} \end{equation*}$$

where $d( {i,K} )$ is the average distance between strain *i* and all other strains from the same cluster (species) *K*; and $d( {i,J} )$ is the average distance between *i* and all strains from the closest distinct cluster (species) *J ≠ K*. By definition, the score *s_i_* is zero if there are no other strains in the same cluster (species) *K*. In our context, the chosen distance is the patristic distance and the clusters are the species as described in the database. The patristic distance between two strains is the total path (sum of branch lengths) along the phylogenetic tree between the two leaves representing the strains. It corresponds to the cophenetic correlation coefficient in cluster analysis (13).

The tree silhouette score as described above does not represent the phylogenetic information fully, since very short branches for sister strains (from the same species) will lead to very high scores but will have less information than one with longer terminal branches. Therefore, we also calculated silhouette scores using a simplified version of the patristic distance, sometimes called ‘path difference’, which neglects estimated branch lengths and just gives the number of internal nodes between two leaves in a tree. The fraction of strains with a positive value for this simplified score is given in Supplementary Figure S1 and estimates the fraction of strains that are closer to another strain in the same species than to one from a different species.

Besides the silhouette scores, we also implemented two statistics explicitly based on the monophyletic status of the species: the monophyly score and the best monophyletic clade score. The monophyly score of a species is the fraction of strains below its last common ancestor that are in fact from this species. In other words, for each species we find the most recent common ancestor among all strains from that species, and then we see if there are also other species below this ancestor on the tree. The score based on the most diverse monophyletic clade is the average patristic distance between all samples below monophyletic clades. A monophyletic clade is one for which all leaves below it belong to the same species, and the average patristic distance between these leaves estimates its phylogenetic divergence. If, for a given species, we found several monophyletic clades, we chose the most diverse one, i.e. the one with the highest average distances. Higher values represent more phylogenetic information (more substitutions per site) while maintaining taxonomic resolution. In both cases we minimized spurious clades by midpoint-rerooting the trees. Note that this ‘best monophyletic clade’ is similar, but not identical to the ‘largest taxonomically consistent subtree’ (LTCS) described by (14). For paraphyletic clades, we also tried using an average instead of the maximum between monophyletic subclades, with very similar results (Supplementary Figure S3).

To have an idea of the distribution of scores in the absence of phylogenetic signal, we generated a random tree with the same leaf names (i.e. same taxonomic information) and same tree length as the original data set. Generation of random tree branch order and lengths was achieved under a simple coalescent model using the Dendropy package version 4.4.0 (15). The same software was used for all tree manipulations and patristic distance calculations, and scikit-learn version 0.21.3 was used for the distance-based silhouette score inference.

### Operon multi-copy analysis

To study the influence of paralogy on the rRNA-based classification, we simulated the effect of long-read-based sequencing (operon multi-copy analysis) by extracting all full operons from the best represented *Pseudomonas* and *Staphylococcus* species in the RefSeq database. These were chosen as they are well-studied, clinically important pathogens. The operons were constructed by finding all annotated 16S, 23S and 5S genes in the genome and merging all genes that were closer than 1000 bp from each other into a single operon. For example, if the first base of a 23S gene was <1000 bp downstream of a 16S gene, then they would belong to the same operon, together with all sites between them. In this way, we reconstructed the 16S-ITS-23S-5S operon that represents the maximum resolution achievable under real-world conditions.

Similarly to the previous analysis, we selected all samples from the RefSeq database (accessed on 15 February 2019) and used the GTDB to establish their taxonomy to the strain level. For this analysis, however, we downsampled more aggressively to an average of two samples per species as we just want to find examples where distinct copies provide conflicting classification information. Our final data sets comprised 45 genomes from *Staphylococcus* and 149 for *Pseudomonas*. Since we included all copies (average of 4.18 operons per *Staphylococcus* and 5.03 for *Pseudomonas* samples), this gave rise to 188 rRNA operon sequences for *Staphylococcus* and 749 rRNA operon sequences for *Pseudomonas*. More information can be found as Supplementary Tables 3 and 4. The operon lengths were between 4887 and 6211 bp for the *Staphylococcus* samples and between 4957 and 7127 bp for *Pseudomonas*. We then aligned all sequences from each genus independently with MAFFT v7.310 (16) (automatic algorithm selection and offset of 0.3) and estimated their maximum likelihood trees with IQTREE v1.6.12 (17) using the HKY+gamma evolutionary model, replicating best practice (18). The alignments had 6789 columns for *Staphylococcus* and 8410 columns for the *Pseudomonas* data set. The scripts necessary for reproducing all results are available as Jupyter notebooks from https://github.com/quadram-institute-bioscience/70S-resolution, and all source code is available under the GNU GPL 3 open source licence.

## RESULTS

By analyzing all paralogous copies at once, we found that even when the full ribosomal RNA operon was available without chimerism or other assembly artifacts, the choice of which genomic copy to analyze affected the phylogenetic inferences. This phenomenon was observed not only at the strain level, but also at the species level as well. The fact that the choice of full-length rRNA gene copy has such a strong influence reinforces the importance of capturing sufficient genetic variability to inform strain-level specificity; this cannot be properly addressed using ribosomal amplicon-based sequencing. Accounting for underlying genetic diversity has implications not only for taxonomic classification, but also for downstream analyses at the ecological or evolutionary level. In this study, we show that this has not been fully explored previously.

To quantify the evolutionary information from distinct rRNA segments we designed a tree silhouette score, which is a measure of how well an evolutionary tree represents taxonomically related strains, and we also used other monophyly-based scores for comparison. Using a comprehensive data set of clinically relevant bacteria, we were able to compare the advantages and disadvantages of analyzing only hypervariable regions of the 16S gene or using all three genes on the ribosomal RNA operon (16S, 23S, 5S), either independently or concatenated in all possible combinations (e.g. ‘16S23S’ represents the aligned sequences from the 16S and 23S genes concatenated for each strain). Since there can be several copies of each operon in the same genome, we used the longest copy in our analysis, to emulate the best case scenario, being conservative while avoiding incomplete operons. However, the results were very similar when we used a consensus of all copies (see ‘Materials and Methods’ section).

### Influence of operon length

**Figure 1.**
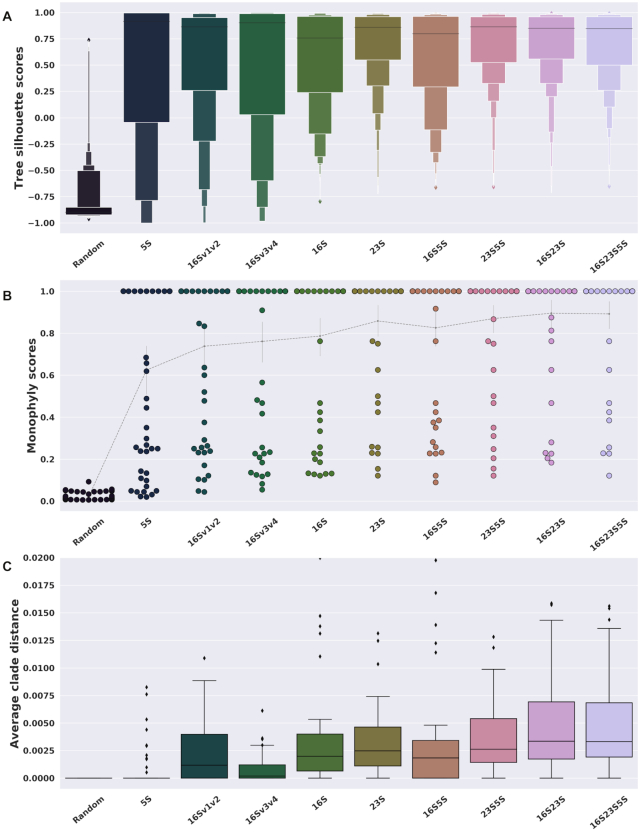
Silhouette and monophyly scores. Results from maximum likelihood trees using the longest copy of the operon. This shows the changes in phylogenetic and taxonomic resolution apparent when either: (i) just a fragment of a gene (regions v1+v2 or v3+v4 of the 16S rRNA), (ii) a whole gene or (iii) several concatenated genes are analyzed. (**A**) The silhouette score describes how close each strain is to others from the same species, compared to the closest strain from a different species. (**B**) Monophyly scores are the fraction of strains from the same species below their last common ancestor. (**C**) The average patristic distance between monophyletic strains is the average distance between strains below the most diverse monophyletic clade of each species. The *Y*-axis is truncated at 0.02.

In cluster analysis, the silhouette score averaged over all strains is used as an indication of clustering fitness (19). However, we can also look at the score for each individual strain, and here we describe a simple ‘tree silhouette score’ using the patristic distance (i.e. path length between strains in the estimated phylogeny). This allowed us to quantify how well the tree retained the taxonomic classification at the species level while also providing evolutionary information. The distributions of silhouette scores for maximum likelihood trees estimated using (i) hypervariable regions of the 16S gene, (ii) entire genes independently and (iii) concatenated gene sequences can be seen in Figure 1A. We also generated a random tree with the same leaf names (same taxonomic information) for comparison. All data sets [(i), (ii) and (iii)] performed in a similar way in terms of taxonomic resolution that could be achieved, with the exception of the 5S gene, that showed an overall poor congruence with the established taxonomic information. We observe a steady increase in the tree silhouette score from shorter to larger segment concatenations. For instance, the 16Sv3v4 segment had high scores for a few strains, but it also provided quite poor scores for several other strains. By computing the proportion of samples with ‘good’ scores (i.e. above zero, which indicates correct clustering), it was apparent that longer sequences led to better taxonomic resolution overall (Supplementary Figure S1).

We also calculated the monophyletic status of a species directly by looking at the last common ancestor of all strains from each species, and seeing how often those ancestors have descendants from other species. The distribution of monophyly scores for all species is shown in Figure 1B; longer sequences resulted in phylogenetic inferences more consistent with the taxonomic classification. In particular, the concatenated data set 16S23S5S has the lowest number of species with a monophyly score <0.9; this value means that, from the most recent common ancestor of a species, for every ten strains nine will be from one species and one will be from a different species. For the longest concatenated data sets, the estimated evolutionary distance between samples from the same species in monophyletic clades was larger than when using smaller sequences (Figure 1C). Overall this means that longer alignments can provide richer phylogenetic signals (more diverse sequences) than short alignments, without compromising taxonomic resolution.

### Influence of distinct operon copies

Species of bacteria often contain multiple copies of the ribosomal operon, and each has its own distinct phylogenetic diversity; intragenomic variability in the 16S gene has been studied most commonly (20,21), but there are also studies on the 23S and 5S genes (22,23). When we consider the evolutionary history of all copies of the ribosomal RNA operons from a given species, one underlying assumption is that all those copies should be monophyletic, i.e. they should be closer to each other than to operons from other species. This is the justification for using a single copy, or a consensus of all operons as sufficient for phylogenetic inference and reconstruction of the correct groupings. However, this is not the case as ribosomal gene copies can be mobile between operons. Sequence variation among ribosomal operons from within one bacterium can be greater than inter-species variation (7).

**Figure 2.**
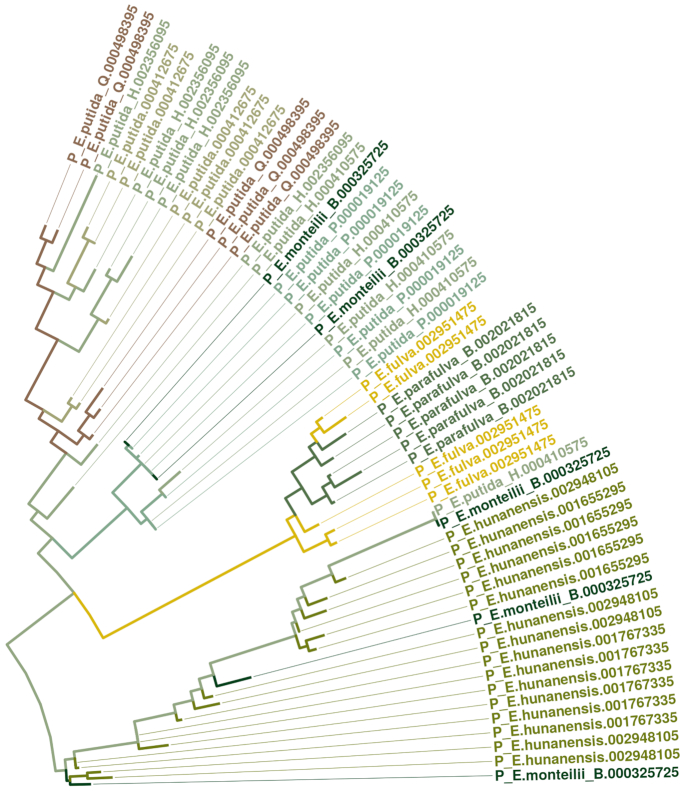
Maximum likelihood tree of full operons for *Pseudomonas*, including paralogs, with colors representing species. Most leaves were pruned, and only the region of interest is shown to emphasize the uncertain location of *P. monteilii_B* (strain *000325725*) and *P. putida_H* (strains *000410575* and *002356095*): phylogeny would vary depending on the copy selected.

The choice of operon affects the phylogeny at the strain level, i.e. the inference of the closest sister taxon is dependent on the choice of the paralog. As an example, each copy of the rRNA operon within a particular *Pseudomonas monteilii_B* strain (labeled *000325725*), clustered with a distinct strain of *P. hunanensis* or *P. putida_H* (Figure 2). The other strains of *P. monteilii_B* clustered together with *P. monteilii* (GTDB assigns an alphabetic suffix to paraphyletic groups according to their reference tree, so in this case *P. monteilii_B* is classified as a distinct species from *P. monteilii*). This behavior is quite common; in the same figure (Figure 2), we observe that the distinct strains of *P. putida_H* (labeled *000410575* and *002356095*) do not form monophyletic groups, being closer to *P. putida_Q* or *P. putida_P*. The same behavior was observed between *P. syringae, P. syringae_M* and *P. cerasi*. Similar results were observed when applying the same analysis with *Staphylococcus*, particularly for *S. hyicus*, *S. lugdunensis* and *S. agnetis*, where the location of certain samples depended on which paralog operon was chosen (Supplementary Figure S4). This agrees with previous studies showing the importance of accounting for the intra-individual diversity of the 16S gene (20,21). Here, we show that this issue cannot be resolved by sequencing larger regions of the genome at the same time. This behavior is not due to stochastic errors in the inference, but to underlying biological processes such as gene duplications in the sequences analyzed (with differential losses) and, to a lesser degree to lateral transfers (24) and incomplete lineage sorting (25).

## DISCUSSION

Our objective was to see if longer rRNA segments offer any advantage over more common 16S markers. From an evolutionary perspective, an informative marker is one that is variable enough to provide diversity within a clade. A pure classification approach, on the other hand, tends to favour measures that provide closer within-clade samples. For this purpose we designed a tree silhouette score that can account for branch lengths, or not, and also a monophyly score accompanied by its distance score. Our overall results show that we do observe an increase in the phylogenetic information—longer alignments are better for affordable population and selection studies—without compromising its taxonomic resolution (i.e. they can be used to classify new pathogens or metagenomic samples as is done with 16S or other markers). The improvement is more pronounced when we compare concatenations including 16S or 23S than when we include 5S to the alignment.

We also show how being able to account for all operon copies—a single genome has several paralogous copies of the rRNA genes—do impact the classification and downstream evolutionary studies. Many studies may be hampered by the confounding effect of multiple copies being summarized by one (e.g. through chimerism or consensus base calling). Long-read assemblies are less affected by these misassemblies, and we will soon need to use all available information from paralogs in deep evolutionary analyses (26,27). The full rRNA operon is a good candidate for long-read studies since it extends the well-known 16S classification advantages while improving the phylogenetic signal. It is worth mentioning that although we use the taxonomy given by the Genome Taxonomy Database (GTDB), the same trends are observed if we use alternative nomenclatures like those provided by SILVA (4) or NCBI (28) (Supplementary Figure S2). Similar results were obtained for alternative choices of samples or genera (results not shown).

Therefore we believe that, whenever possible, longer segments of the rRNA operon should be preferred to 16S, while accounting for the heterogeneity in the copies—both tasks that become feasible with long-read sequencing technologies. This would allow the resulting trees to be interpreted not only for classification, but also potentially for downstream analyses like diversity or divergence times estimation, where 16S currently presents limited usefulness (29).

## DATA AVAILABILITY

The source code for reproducing all analyses is available under the GNU GPL 3 open source licence at https://github.com/quadram-institute-bioscience/70S-resolution.

## Supplementary Material

lqz016_Supplemental_Files
