# Supplementary material for: Taxonomic resolution of the ribosomal RNA operon in bacteria: implications for its use with long-read sequencing
Source: NAR Genom Bioinform. 2019 Nov 14;2(1):lqz016. doi: 10.1093/nargab/lqz016 (PMC7671355; doi:10.1093/nargab/lqz016)
Supplement: lqz016_Supplemental_Files [file lqz016_supplemental_files.zip › 201909rRNAresolutionManuscript-SupplFiguresTables.pdf]

Supplementary Material (Figures and Tables) for

Taxonomic resolution of the ribosomal RNA operon in bacteria: Implications for its use with long read sequencing

Leonardo de Oliveira Martins*, Andrew J. Page, Ian G. Charles

Quadram Institute Bioscience, Norwich Research Park, Norwich, NR4 7UQ, UK.

*Corresponding Author: email: Leonardo.de-Oliveira-Martins@quadram.ac.uk

All supplementary material, including the tables, and Jupyter notebooks with all analyses can be found at https://github.com/quadram-institute-bioscience/70S-resolution.

Supplementary Tables

Supplementary tables 1, 2, and 3 are provided as CSV tables in the github repository.

Supplementary Table 1 (description): Accession numbers, species names, strain names, and summary statistics for the 691 samples used in the single-copy analysis of the ribosomal RNA sequences. The statistics shown are the number of copies, length of longest copy, and length of consensus for each of the three rRNA genes. The size of the consensus sequence is usually larger than the longest sequence since it represents an alignment of all copies (where insertions/deletions are introduced). The CSV file can be found in https://github.com/quadram-institute-bioscience/70S-resolution/026_results/all_single_copy.csv

Supplementary Table 2 (description): Accession numbers, species names, strain names and statistics for the 149 *Pseudomonas* genomes used in the operon analysis (the alignment has 749 sequences of 8410 sites in total). The statistics are the number of operons, as well as the minimum, mean, and maximum operon lengths. The mean operon size before alignment is 5215 ± 126 sites, with min=4957 and max=7127 sites. The CSV file can be found in https://github.com/quadram-institute-bioscience/70S-resolution/024_results/Pseudomonas.csv

Supplementary Table 3 (description): Accession numbers, species names, strain names and statistics for the 45 *Staphylococcus* genomes used in the operon analysis (the alignment has 188 sequences of 6789 sites in total). The statistics are the number of operons, as well as the minimum, mean, and maximum operon lengths. The mean operon size before alignment is 5119 ± 289 sites, with min=4887 and max=6211 sites. The CSV file can be found in https://github.com/quadram-institute-bioscience/70S-resolution/024_results/Staphylococcus.csv

Supplementary Table 4: Gene length statistics over samples from single-copy analysis.

		mean	std	min	5%	95%	max
**16S**	Longest	1550	32	1110	1540	1566	1569
	Consensus	1556	46	1514	1544	1567	2741
**23S**	Longest	2908	66	2070	2901	2930	3040
	Consensus	2924	89	2833	2902	2938	5180
**5S**	Longest	116	0.8	105	115	116	127
	Consensus	117	32	115	115	116	960

Supplementary Figures

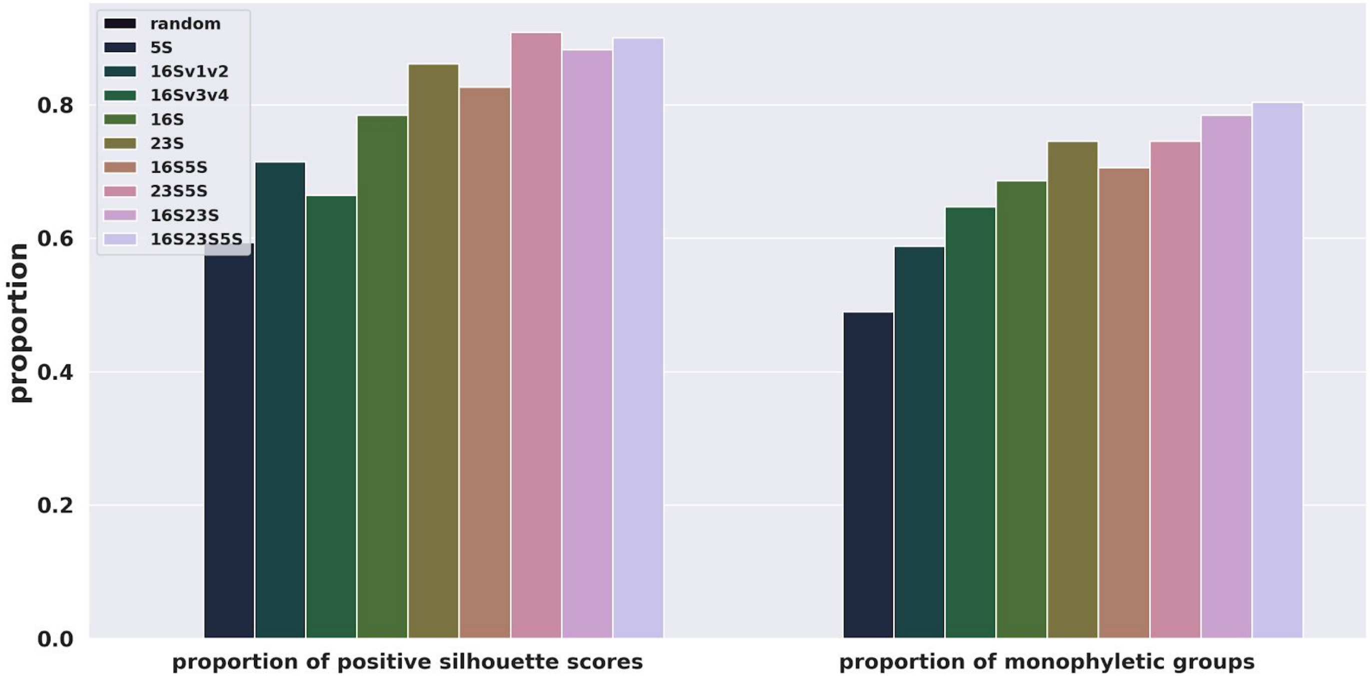

Supplementary Figure S1: Proportion of samples with positive silhouette scores (using the simplified distance) and proportion of monophyletic species, according to maximum likelihood trees estimated from distinct combinations of rRNA sequences.

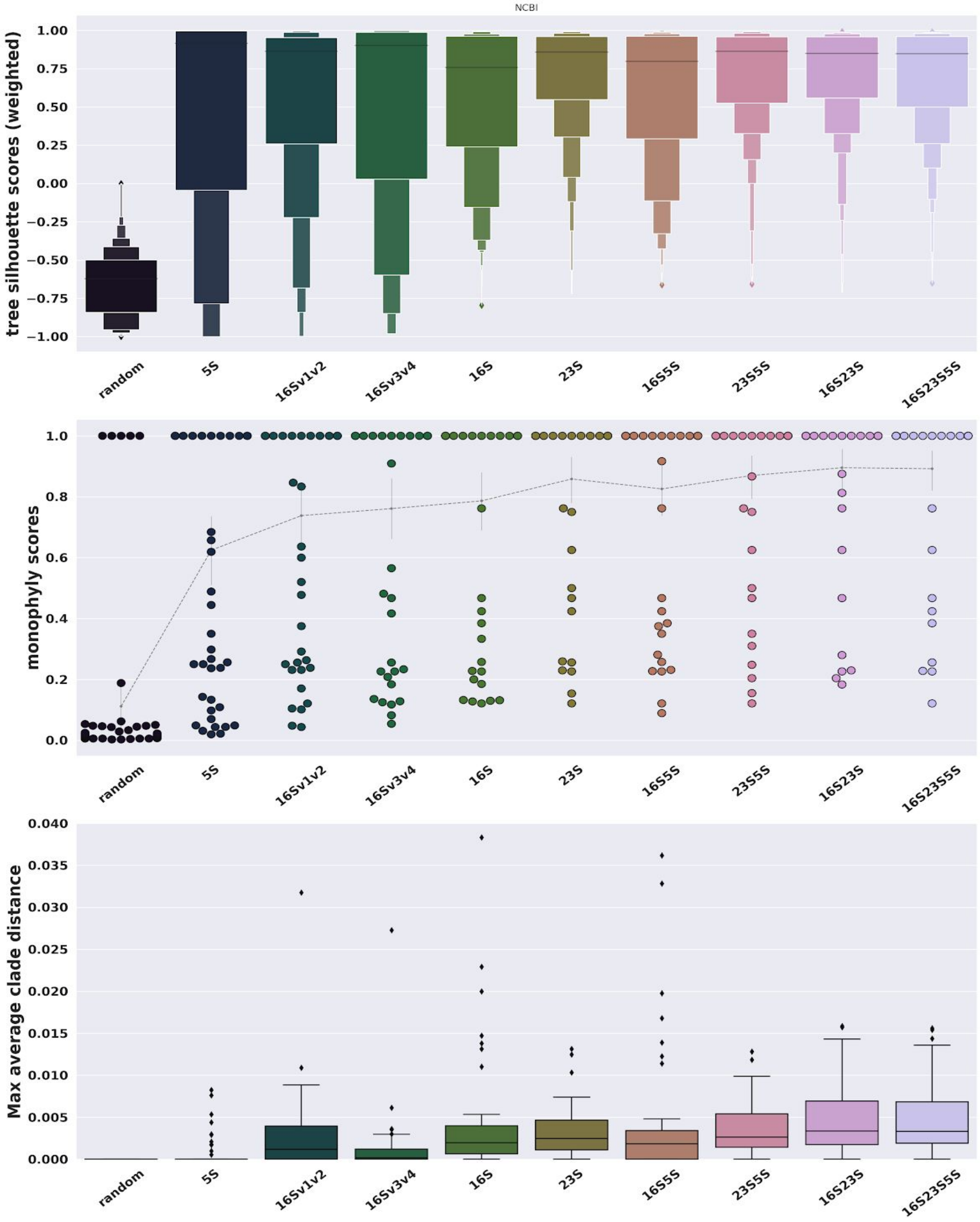

Supplementary Figure S2: Silhouette and monophyly scores for the same samples (sequences and trees) as main analysis, but using an alternative taxonomy, from NCBI. ML trees of the longest copy of the operon. It shows changes in phylogenetic and taxonomic resolution for: i) just a fragment of a gene (regions v1+v2 or v3+v4 of the 16S rRNA); ii) a whole gene; or iii) several concatenated genes are analysed. The silhouette score is at the top; it describes how close each strain is to others from the same species, compared to the closest strain from a different species. Monophyly scores are in the middle panel; they are the fraction of strains from the same species below their last common ancestor. The average patristic distance between the “best” monophyletic strains is shown at the bottom; it is the average distance between strains below the most diverse monophyletic clade of each species.

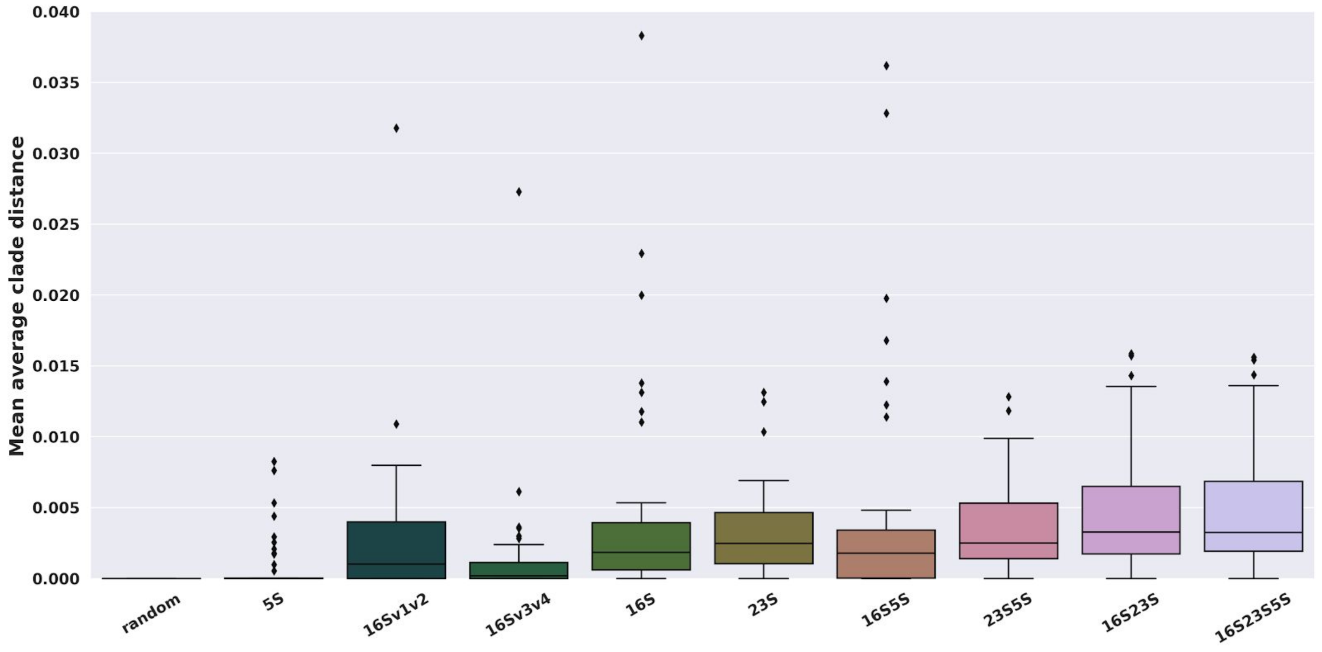

Supplementary Figure S3: The average patristic distance between all monophyletic subtrees for every species, for the same samples and binomial nomenclature as Suppl Fig 2 above. We use the NCBI taxonomy to emphasise the similarity to the Suppl Fig 2 above, but the same results were observed if we use the GTDB or SILVA taxonomic classifications of these samples.

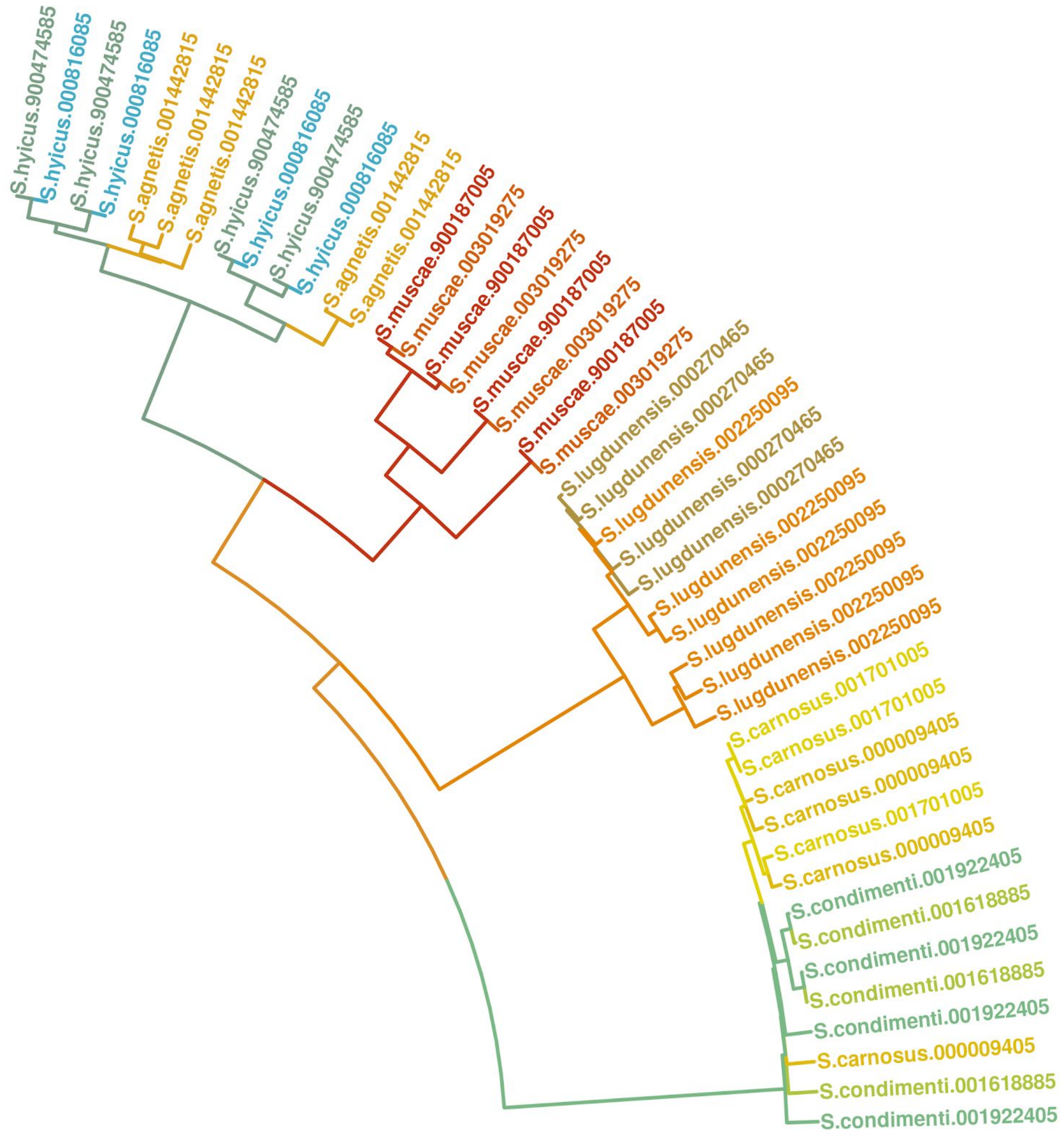

Supplementary Figure S4: Maximum likelihood tree of full operons for Staphylococcus samples, including paralogs, with colours representing strains. This tree has most leaves pruned, and only the region of interest is shown to emphasise the uncertain locations
